# Prevalence and diversity of *Aphanomyces astaci* in cambarid crayfish of Pennsylvania: where native and introduced hosts meet

**DOI:** 10.1017/S0031182025000022

**Published:** 2025-01

**Authors:** Adam Petrusek, Michaela Mojžišová, Adéla Mikešová, Radka Piálková, David A. Lieb

**Affiliations:** 1 Department of Ecology, Faculty of Science, Charles University, Prague, Czechia; 2 Department of Medical Biology, Faculty of Science, University of South Bohemia in České Budějovice, České Budějovice, Czechia; 3 Department of Zoology, Faculty of Science, University of South Bohemia in České Budějovice, České Budějovice, Czechia; 4 Pennsylvania Fish and Boat Commission, Bellefonte, PA, USA

**Keywords:** crayfish plague, genotyping, haplogroups, host specificity, native hosts

## Abstract

The crayfish plague pathogen *Aphanomyces astaci* (Oomycota: Saprolegniales) is native to North America but expanded with its crayfish hosts to other regions. In most of its invaded range, *A. astaci* haplotypes are associated with specific American crayfish, probably due to introduction bottlenecks, but haplotype diversity is higher and clear host-specific associations are lacking in its native range. However, little is known about the infection rate and load of this pathogen in North America. We investigated the distribution, prevalence and genetic variation of *A. astaci* in Pennsylvania (eastern USA), where multiple native and introduced crayfish species (family Cambaridae) occur. We used *A. astaci*-specific quantitative PCR to screen 533 individuals representing 8 crayfish species (2 *Cambarus* and 6 *Faxonius*) from 49 sites. *Faxonius limosus*, an American species first introduced to Europe and carrier of *A. astaci* genotype group E, was of particular interest. We confirmed *A. astaci* infections in 76% of sites in all but 1 host taxon, with the pathogen infection rate and load comparable to established populations of North American crayfish studied in Europe and Japan. Despite the absence of highly infected hosts, we genotyped *A. astaci* from 14 sites. We only detected 2 mitochondrial haplotypes, but nuclear markers indicated the presence of at least 4 distinct pathogen genotypes, none documented from invaded areas in Europe or Asia. Genotype group E was not detected in *F. limosus*, possibly due to limited spatial distribution of the original strain. Our results highlight both benefits and limitations of combining multiple pathogen genotyping methods.

## Graphical abstract

**Figure:**
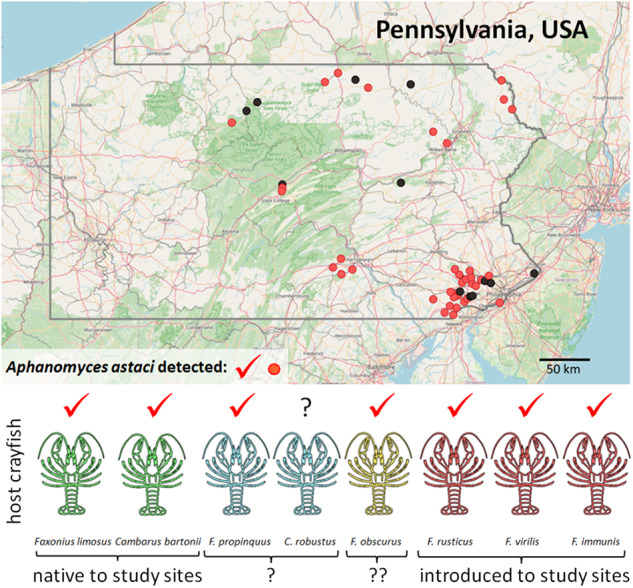

## Introduction

The oomycete *Aphanomyces astaci* (Saprolegniales), the causative agent of crayfish plague, has been introduced from North America to several regions across the globe, where it threatens native crayfish populations or aquaculture production of species susceptible to this disease. Crayfish plague has a particularly strong impact in Europe, where it has been causing mass mortalities of local crayfish since the 19th century (Alderman, 1996; Holdich et al., 2009), and in Japan where it has been implicated in the decline of the endemic Japanese crayfish *Cambaroides japonicus* (Martín-Torrijos et al., 2018). Although the original mode of introduction of this pathogen to Europe remains unclear, most *A. astaci* strains documented in its invaded range were associated with introductions of particular non-native crayfish species of North American origin (Martín-Torrijos et al., 2018; Ungureanu et al., 2020). These crayfish usually serve as asymptomatic hosts of *A. astaci*, thanks to their long coevolutionary history with this pathogen.

Despite the coevolution, however, *A. astaci* is not a harmless commensal for its original hosts, as it rather behaves as an opportunistic pathogen. Its hyphae penetrating through the cuticle are stopped by an active response of the host’s immune system and encapsulated by deposited melanin (Cerenius et al., 2003), hosts from infected populations may show gross symptoms such as limb loss or visible melanized lesions on the body surface (e.g. Jussila et al., 2016), and even otherwise non-symptomatic hosts may die with symptoms of acute crayfish plague when exposed to high doses of *A. astaci* spores or stressed (Diéguez-Uribeondo and Söderhäll, 1993; Kozubíková et al., 2011a; Aydin et al., 2014). Furthermore, *A. astaci* may interact with other pathogens, resulting in further detrimental impacts to the hosts (Edsman et al., 2015). Upon contact, North American crayfish infected with *A. astaci* may transfer it to species susceptible to crayfish plague (reviewed in Svoboda et al., 2017). Outside the native range of both pathogen and hosts, cases of horizontal transfer of *A. astaci* strains between different North American crayfish have also been occasionally documented (James et al., 2017; Mojžišová et al., 2022, 2024).

Most mass crayfish mortalities caused by *A. astaci* in Europe have been associated with 4 major pathogen genotype groups (labelled by capital letters A, B, D and E; see Svoboda et al., 2017; Ungureanu et al., 2020 for review). Group A has been spreading through the continent since the 19th century without a specific original host. Groups B and D were introduced only after the mid-20th century with their highly invasive hosts, the signal crayfish *Pacifastacus leniusculus* and the red swamp crayfish *Procambarus clarkii*, respectively. These introductions likely happened repeatedly, as both host species were introduced to Europe for fisheries and aquaculture purposes in high numbers and multiple times (Henttonen and Huner, 1999; Gherardi, 2006). The third widespread North American crayfish invader in Europe, the spiny-cheek crayfish *Faxonius limosus*, the original host of *A. astaci* group E (Kozubíková et al., 2011a), however, has a different history, with apparently a single successful introduction in 1890 (Filipová et al., 2011). Apart from the above-mentioned *A. astaci* genotype groups isolated to axenic cultures, additional strains of the pathogen introduced to Europe have been documented, either from mass mortalities or chronic infections of native crayfish (Grandjean et al., 2014; Panteleit et al., 2018; Mojžišová et al., 2020), or from crayfish traded for ornamental purposes and subsequently released to the wild (Mojžišová et al., 2024).

The prevalence of *A. astaci* (i.e. the infection rate estimated by molecular detection methods) in its invasive host populations has been studied frequently in various countries where crayfish plague threatens native species (e.g. Kozubíková et al., 2011b; Grandjean et al., 2017; Mrugała et al., 2017; Laffitte et al., 2024). In contrast, much less attention has been paid to the distribution and diversity of *A. astaci* in its native range, North America, with no more than 5 studies available so far, 4 from the USA (Makkonen et al., 2019; Panteleit et al., 2019; Butler et al., 2020; Martín-Torrijos et al., 2021b) and 1 from Mexico (Martín-Torrijos et al., 2023). A summary of the evidence from the USA (Martín-Torrijos et al., 2021b) indicates, not surprisingly, a substantially higher diversity (i.e. presence of more mitochondrial haplotypes) of *A. astaci* in local crayfish hosts than across the Atlantic. The results also suggest that the association between particular host species and pathogen haplogroups in the USA is weaker, if present at all. However, the presence of matching *A. astaci* genotypes or haplogroups were confirmed in populations of *P. clarkii* and *P. leniusculus* from their source regions in the USA and from regions in Europe colonized by those species (Makkonen et al., 2019; Martín-Torrijos et al., 2021b). Data on the *A. astaci* infection rate in native North American crayfish populations are lacking altogether. Panteleit et al. (2019) applied quantitative PCR to screen for *A. astaci* in several US populations of the rusty crayfish *Faxonius rusticus*, but their study focused on the non-native range of this species, which is a widespread invader in North American waters (Durland Donahou et al., 2024).

The aim of our study was to investigate the distribution, prevalence and diversity of *A. astaci* in Pennsylvania, a US state where multiple potential host species, both native to the region and introduced from other parts of the USA, come into contact (Lieb et al., 2011a, 2011b). Among the native taxa, *F. limosus* deserves special attention as the first successfully established North American crayfish in Europe, with which *A. astaci* genotype group E was co-introduced (Kozubíková et al., 2011a; Ungureanu et al., 2020). The Delaware River basin, which covers eastern Pennsylvania, was assumed to be the source of the only successful introduction of *F. limosus* to Europe (Schikora, 1916; Henttonen and Huner, 1999), but phylogeographic data suggest that, at least for the Lower Delaware watershed, this is unlikely (Filipová et al., 2011). The presence of *A. astaci* was confirmed in 3 non-native crayfish species in a small area of Pennsylvania (Lancaster County, Lower Susquehanna watershed) by Butler et al. (2020), and axenic cultures of a strain assigned by these authors to *A. astaci* genotype group C were isolated from one of them, the Allegheny crayfish *Faxonius obscurus*.

We hypothesized that our broader screening in Pennsylvania would confirm a widespread presence of *A. astaci* in both introduced and native crayfish species, and that infection rates of this pathogen in host populations would vary substantially, corresponding to patterns documented from areas invaded by *A. astaci* hosts in other continents. For *A. astaci*-positive samples, we attempted to apply several complementary genotyping methods to differentiate between the pathogen strains involved. Considering the generally low infection loads usually observed in non-symptomatic *A. astaci* hosts (e.g. Tilmans et al., 2014; Panteleit et al., 2019; Mojžišová et al., 2024), we expected that only a small fraction of such samples would allow for successful pathogen genotyping, but that we would nevertheless detect a substantial variation of the pathogen in our study region, with likely presence of yet uncharacterized *A. astaci* genotypes. Out of the already known ones, we expected to encounter *A. astaci* group E in *F. limosus*, and the genotype already reported by Butler et al. (2020) from *F. obscurus*. For the latter, we wanted to clarify its identity in respect to *A. astaci* group C, originally isolated from an entirely unrelated host (*P. leniusculus*) originating from a lake at the Pacific coast of British Columbia.

## Materials and methods

### Crayfish sampling

**Figure 1.**
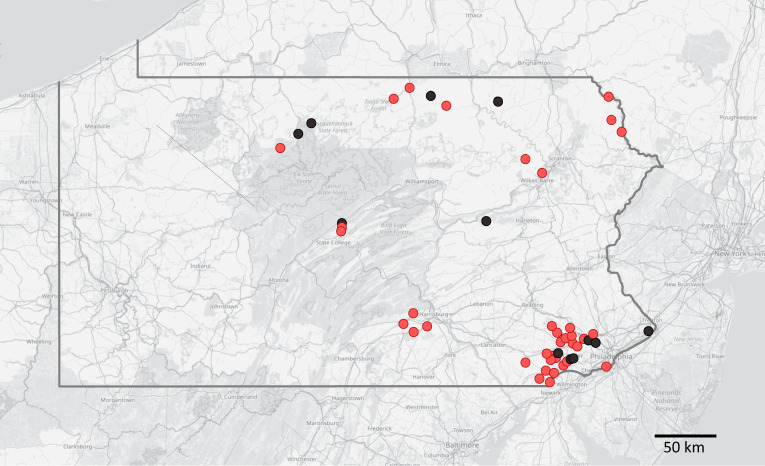
Distribution of studied localities in Pennsylvania (with state borders highlighted), showing detection of the crayfish plague pathogen in crayfish populations (confirmed *A. astaci* presence in light red, no detection in black). Background map is based on openstreetmap.

Crayfish were opportunistically sampled between 2017 and 2022 in central to eastern Pennsylvania (Figure 1) from various lotic as well as lentic aquatic habitats, including small and large streams, rivers, ponds and lakes, as a part of a larger effort to survey the Susquehanna and Delaware River drainages of Pennsylvania for crayfish. Additional specimens were collected from streams in southeastern Pennsylvania during targeted efforts to document the distribution of one of the state’s rarest crayfish species, an undescribed member of the *Cambarus acuminatus* complex (Lieb et al., 2008, 2011a, 2011b; Williams et al., 2020). Crayfish from small streams (<1 m wide), as well as from nearshore habitats of standing waters, were sampled with dip nets (41 × 38 cm) for ca 1-person hour; all available bottom substrates including root masses and aquatic vegetation were covered. In wider streams, crayfish were sampled using weighted seine nets stretched across the bottom, upstream of which the substrate was disturbed; 10 seine hauls were performed in ca 125-m reach of each stream.

Distribution of studied localities in Pennsylvania

Crayfish were identified to species by morphological characteristics given in Swecker et al. (2010) and Thoma (2022). The number of processed crayfish specimens depended on local population density and capture success, and ranged from 1 to 30 per site and species. Altogether, 533 host individuals from 49 sites were analysed (Table 1, Figures 1 and S1), representing 8 host crayfish taxa (for their common names, see Table 2): 2 certainly native to the region (*Cambarus bartonii, F. limosus*), 2 additional ones also likely native (*Cambarus robustus, Faxonius propinquus*), 3 certainly introduced (*Faxonius immunis, F. rusticus, F. virilis*) and 1 likely introduced to most, if not all sampled sites (*F. obscurus*). At 14 localities, coexisting crayfish species were collected (usually 2, in 1 case 3), in various combinations (Table 1).

**Table 1.** List of studied localities and summary of *A*. *astaci* detection and genotyping (separate per species and site). Results of *A*. *astaci* detection for each individual are included in supplementary Table S1, detailed genotyping results, including variation at microsatellite markers, are provided in Table 3

No: Locality [basin]^a^	Latitude	Longitude	Sampling month	Crayfish sp.	Status^b^	No. inf /total	Prevalence (CI^c^)	Agent level^d^	No. genotyped	mtDNA/qPCR^e^
1: East Branch White Clay Creek [D]	39.8499	−75.7838	Aug 2018	*F. limosus*	N	5/8	63% (24–91%)	A2–A3		
2: Chester Creek [D]	39.9314	−75.5506	Aug 2018	*F. limosus*	N	0/6	0% (0–46%)	N/A		
3: West Branch Red Clay Creek [D]	39.8306	−75.7217	Aug 2018	*F. limosus*	N	3/6	50% (12–88%)	A2–A3		
4: West Branch White Clay Creek [D]	39.7853	−75.8564	Aug 2018	*F. limosus*	N	7/7	100% (59–100%)	A2–A3	3	a/C
5: Broad Run #2 [D]	39.9447	−75.7012	Aug 2018	*C. bartonii*	N	2/2	100%	A2		
				*F. limosus*	N	2/2	100%	A2–A3		
6: Broad Run #1 [D]	39.9517	−75.7257	Aug 2018	*F. limosus*	N	1/3	33%	A3		
7: Broad Run at bridge [D]	39.7722	−75.7461	Aug 2018	*F. limosus*	N	1/ 7	14% (0.4–58%)	A2		
8: Furnace Run [S]	40.9584	−76.3845	Sep 2018	*C. bartonii*	N	0/4	0%	N/A		
9: French Creek, downstream [D]	40.1769	−75.7314	Sep 2017	*F. limosus*	N	0/1	0%	N/A		
				*F. rusticus*	I	4/4	100%	A2–A3		
				*C. bartonii*	N	1/1	100%	A2		
10: Crooked Creek [S]	41.9217	−77.1323	Nov 2021	*F. propinquus*	N?	6/6	100% (54–100%)	A2		
11: Crooked Creek [S]	41.8424	−77.2765	Nov 2021	*F. obscurus*	I?	2/9	22% (3–60%)	A2		
12: Delaware River [D]	41.6106	−75.0672	Aug 2021	*F. limosus*	N	6/13	46% (19–75%)	A2–A3		
13: Delaware River [D]	41.8670	−75.1934	Aug 2021	*F. limosus*	N	2/2	100%	A2–A3		
14: Leonard Creek [S]	41.4134	−76.0012	Aug 2021	*C. bartonii*	N	4/6	67% (22–96%)	A2–A3		
				*F. propinquus*	N?	3/4	75%	A2		
15: North Branch Calkins Creek [D]	41.6993	−75.1658	Aug 2021	*F. rusticus*	I	7/7	100% (59–100%)	A2–A3	1	a/C
16: North Branch Sugar Creek [S]	41.7957	−76.7753	Nov 2021	*F. obscurus*	I?	5/8	63% (24–91%)	A2–A3		
17: Freeman Run [S]	41.6699	−78.0885	Nov 2021	*C. bartonii*	N	0/1	0%	N/A		
				*C. robustus*	N?	0/2	0%	N/A		
18: Spring Creek [S]	40.9388	−77.7889	Oct 2021	*C. bartonii*	N	0/9	0% (0–34%)	N/A		
19: Spring Creek [S]	40.8833	−77.7863	Oct 2021	*F. rusticus*	I	7/14	50% (23–77%)	A2–A3		
20: Spring Creek [S]	40.9093	−77.7855	Oct 2021	*F. obscurus*	I?	1/7	14% (0.4–58%)	A2		
21: West Creek [S]	41.4825	−78.3791	Nov 2021	*C. robustus*	N?	0/1	0%	N/A		
				*F. obscurus*	I?	1/2	50%	A2		
22: Abrahams Creek [S]	41.3135	−75.8392	Aug 2021	*F. propinquus*	N?	5/10	50% (19–81%)	A2–A3	1	**b/C**
23: South Creek [S]	41.8313	−76.2745	Aug 2021	*C. bartonii*	N	0/9	0% (0–34%)	N/A		
24: Cowley Run [S]	41.5904	−78.1984	Nov 2021	*C. robustus*	N?	0/1	0%	N/A		
25: unnamed tributary to Pickering Creek [D]	40.0980	−75.5441	Sep 2021	*C. bartonii*	N	1/1	100%	A3	1	a/?
				*F. virilis*	I	2/2	100%	A2–A3		
26: Mill Creek [S]	41.8729	−76.9148	Nov 2021	*C. bartonii*	N	0/1	0%	N/A		
				*F. obscurus*	I?	0/1	0%	N/A		
27: Radley Run [D]	39.9159	−75.5943	Jul 2022	*C. bartonii*	N	6/6	100% (54–100%)	A2–A3	1	?/C
				*F. limosus*	N	5/5	100% (48–100%)	A2–A3		
28: Bennetts Run [D]	39.8875	−75.6287	Jul 2022	*C. bartonii*	N	3/5	60% (15–95%)	A2–A4	2	a/C
				*F. limosus*	N	4/7	57% (18–90%)	A2–A3	1	a/C
29: Green Tree Run [D]	40.0323	−75.4912	Jul 2022	*C. bartonii*	N	1/2	50%	A4	1	**a/B**
30: Van Sciver Lake [D]	40.1431	−74.8070	Jun 2022	*F. limosus*	N	0/1	0%	N/A		
31: Schuylkill River [D]	40.1109	−75.3445	Jul 2022	*F. rusticus*	I	8/29	28% (13–47%)	A2–A3	1	a/C
32: Arrowmink Creek [D]	40.0577	−75.3098	Jul 2022	*F. virilis*	I	0/30	0% (0–12%)	N/A		
33: Crowe Creek [D]	40.0755	−75.3854	Jul 2022	*C. bartonii*	N	0/6	0% (0–46%)	N/A		
34: unnamed tributary of West Branch Brandywine Creek [D]	39.9610	−75.7757	Jul 2022	*C. bartonii*	N	1/17	6% (0.2–29%)	A4	1	a/C
35: Pine Creek #2 [D]	40.0823	−75.6136	Jul 2022	*F. virilis*	I	17/17	100% (80–100%)	A2–A3	3	a/C
36: Pine Creek #1 [D]	40.0605	−75.6424	Jul 2022	*F. immunis*	I	1/6	17% (0.4–64%)	A2		
37: East Branch Brandywine Creek [D]	39.9686	−75.6732	Aug 2022	*F. limosus*	N	0/10	0% (0–31%)	N/A		
38: unnamed tributary of West Branch Brandywine Creek [D]	39.9245	−75.7374	Jul 2022	*C. bartonii*	N	4/13	31% (9–61%)	A2–A3		
				*F. limosus*	N	1/5	20% (1–72%)	A2		
39: pond at Fort Mifflin [D]	39.8759	−75.2119	Jun 2022	*F. limosus*	N	1/1	100%	A3		
40: East Branch Octoraro Creek [S]	39.9047	−75.9960	Aug 2022	*F. limosus*	N	3/5	60% (15–95%)	A2–A3		
41: Chester Creek [D]	39.9274	−75.5799	Jul 2022	*C. bartonii*	N	0/8	0% (0–37%)	N/A		
42: Birch Run [D]	40.1188	−75.6666	Jul 2022	*C. bartonii*	N	1/17	6% (0.2–29%)	A4	1	a/C
43: Little Valley Creek [D]	40.0475	−75.5288	Aug 2022	*F. virilis*	I	10/24	42% (22–63%)	A2–A3		
44: Yellow Breeches Creek [S]	40.1434	−77.0911	Aug 2022	*C. bartonii*	N	1/1	100%	A2	1	a/C
				*F. rusticus*	I	21/21	100% (84–100%)	A2–A4		
45: unnamed tributary of Trout Creek [D]	40.0843	−75.4190	Jul 2022	*F. virilis*	I	12/15	80% (52–96%)	A2–A3		
46: Letort Spring Run [S]	40.2043	−77.1823	Aug 2022	*C. bartonii*	N	1/23	4% (0.1–22%)	A2		
47: unnamed tributary of Conodoguinet Creek [S]	40.2745	−77.0993	Aug 2022	*F. rusticus*	I	20/25	80% (59–93%)	A2–A4	2	a/C
				*F. virilis*	I	20/21	95% (76–100%)	A2–A3	3	a/C
48: unnamed tributary of Yellow Breeches Creek [S]	40.1811	−76.9491	Aug 2022	*F. rusticus*	I	23/25	92% (74–99%)	A2–A4	1	?/C
				*C. bartonii*	N	2/5	40% (5–85%)	A2–A3		
49: French Creek, upstream [D]	40.1381	−75.5527	Aug 2022	*F. rusticus*	I	10/14	71% (42–92%)	A2–A3		
				*F. virilis*	I	1/2	50%	A3		

a Locality numbers correspond to Supplementary Table 1. Abbreviations of major river basins: [D]: Delaware, [S]: Susquehanna.

b Status of crayfish species at the given site: N: native; I: introduced; ?: status uncertain.

c 95% confidence interval (CI) of the pathogen prevalence was calculated for populations with at least 5 individuals analysed.

d Agent levels are provided for populations with at least 1 *A. astaci*-positive specimen (A2: 5–50 PFU, A3: 50–1000, A4: 1000–10 000 PFU per reaction). N/A: no specimen considered *A. astaci*-positive, i.e. no DNA isolate exceeding agent level A1 (<5 PFU per reaction).

e Genotyping results are provided for mitochondrial ribosomal markers (mtDNA; lower-case letters) according to Makkonen et al. (2018) and for qPCR-based genotyping assays (upper-case letters) after Di Domenico et al. (2021); question marks indicate when a given method was not successful. Unusual marker combinations are highlighted in bold.

**Table 2.** Summary of qPCR-based detection of *A*. *astaci* in studied crayfish. Species are ordered by the number of analysed individuals, in descending order. Spatial distribution of studied populations and their infection status are provided separately for each species in Figure S1

	Populations	Individuals		Genotyping^b^	
Species	*A. astaci-*positive/total [%]		Prevalence range^a^	Individuals	populations
*Faxonius rusticus*	8/8 [100%]	100/139 [72%]	28–100%	6	5
Rusty crayfish					
*Cambarus bartonii*	13/20 [65%]	28/137 [20%]	0–100%	7	6
Appalachian brook crayfish					
*Faxonius virilis*	6/7 [86%]	62/111 [56%]	0–100%	6	2
Virile crayfish					
*Faxonius limosus*	13/17 [76%]	41/89 [46%]	0–100%	4	2
Spiny-cheek crayfish					
*Faxonius obscurus*	4/5 [80%]	9/27 [33%]	0–63%	—	—
Allegheny crayfish					
*Faxonius propinquus*	3/3 [100%]	14/20 [70%]	50–100%	1	1
Northern clearwater crayfish					
*Faxonius immunis*	1/1 [100%]	1/6 [17%]	0–17%	—	—
Calico crayfish					
*Cambarus robustus*	0/3 [0%]	0/4 [0%]	0%	—	—
Big Water crayfish					

a The proportion of *A. astaci*-positive populations and the pathogen prevalence estimates may be biased by sites with a low number of analysed specimens of the given species, as well as by limitations of the molecular detection methods.

b ‘Genotyping’ columns summarize the total number of individuals and populations, from which we obtained at least some genotyping data.

Crayfish individuals were euthanized and preserved in 80% molecular-grade ethanol after sampling. Small specimens were kept whole, larger specimens were dissected before transport to the molecular laboratory of Charles University, Prague, Czechia. Only body parts suitable for screening for *A. astaci* (primarily soft abdominal cuticle and tail fan, i.e. uropods and telson, occasionally pereiopods) were used for DNA isolation.

## A. astaci *detection*

DNA from crayfish tissues was isolated following Oidtmann et al. (2006) and Kozubíková et al. (2008), by grinding cuticle cleaned from muscle and other soft tissues in liquid nitrogen (usually 40–60 mg, smaller amounts for very small specimens) and processing this homogenate with a DNeasy Blood and Tissue Kit (Qiagen, Hilden, Germany). The isolation generally followed the manufacturer’s protocol, the incubation time was at least 12 h at 56 °C, and the elution to 100 μL of AE buffer pre-heated to 65 °C was performed twice to increase DNA yield (reaching the total DNA isolate volume of 200 μL). Two negative controls, consisting of tubes containing 50 μL of sterile water, were included with each isolation batch; one was kept open during mechanical tissue processing, the other while using the isolation kit.

The presence of *A. astaci* DNA was evaluated by quantitative real-time PCR (qPCR), using the TaqMan MGB-based protocol designed to specifically detect the internal transcribed spacer (ITS) region of *A. astaci*. For part of the samples, an assay from Vrålstad et al. (2009), following a slightly modified protocol given in Svoboda et al. (2014), was applied. The bulk of the samples, including part of the *A. astaci*-positive isolates previously tested with the Vrålstad et al. (2009) assay (see Table S1 for individual results), were analysed using the recently published assay of Strand et al. (2023), which has increased specificity over the original protocol. The reaction mix of 25 μL volume was based on TaqMan Environmental Master Mix 2.0 (Applied Biosystems, Waltham, MA, USA) and contained 5 μL of the DNA isolate, primers (AphAstITS_15F, AphAstITS_145R; 500 nM) and TaqMan probe (AphAstITS_61T; 100 nM) according to the original protocol. The reactions were conducted in Bio-Rad iCycler iQ5 (Bio-Rad Laboratories, Hercules, CA, USA), using the EMM^2^ thermal profile of Strand et al. (2023) with the annealing temperature of 58 °C. Data were processed using iQ5 2.1 Standard Edition Optical System Software (Bio-Rad).

In each qPCR reaction, negative controls from the DNA isolation step were included, as well as no-template controls and 4 positive calibrants in duplicates (4-fold dilution series based on the synthetic oligonucleotide of the target ITS sequence, with starting concentration of 5 × 10^5^ PCR-forming units (PFUs) per reaction). These were used to calculate the starting DNA quantities in the analysed samples. The samples were then assigned, according to Vrålstad et al. (2009), to semi-quantitative categories (agent levels), in which the amount of target DNA increases exponentially (see also footnote d of Table 1). Agent levels A0 (no target DNA detection) and A1 (traces of target DNA; less than 5 PFU in the reaction) were considered negative, agent levels from A2 (5–50 PFU) to A4 (10^3^–10^4^ PFU) were considered *A. astaci*-positive. Potential PCR inhibition was evaluated by screening a subset of randomly selected DNA isolates (including those yielding negative results) 10× diluted, and comparing the differences in cycle threshold (Ct) between non-diluted and diluted samples (Kozubíková et al., 2011b).

For localities where at least 5 specimens of a given host species were collected, a 95% confidence interval of *A. astaci* prevalence, based on the detected infection rates, was calculated by the epi.conf function of epiR package v. 2.0.75 (Stevenson and Sergeant, 2024), using R v. 4.3.3. (R Core Team, 2024). Considering the inherent limitation of the molecular detection methods, especially when testing only a limited part of the host crayfish body, but also due to laboratory procedures (such as DNA extraction efficiency), the reported infection rate values including their confidence intervals have to be considered conservative underestimations. However, this bias is analogous to other recent studies using a comparable methodology (e.g. Laffitte et al., 2024; Mojžišová et al., 2024).

## A. astaci *genotyping*

Three complementary genotyping approaches were used to assess the diversity of the crayfish plague pathogen in the studied hosts, as in Mojžišová et al. (2022, 2024). The methods target different markers in the *A. astaci* genome and characterize them by different methodologies, but each of them has been repeatedly successfully applied for mixed genome samples containing DNA of crayfish hosts as well as *A. astaci*. Specifically, we (1) sequenced fragments of 2 mitochondrial genes (following Makkonen et al., 2018); (2) screened by qPCR for presence of 5 different anonymous nuclear markers (following Di Domenico et al., 2021) and (3) attempted to characterize variation at 9 polymorphic microsatellite loci developed for *A. astaci* (following Grandjean et al., 2014). All these methods were originally validated on axenic cultures of strains representing different *A. astaci* genotype groups, but their subsequent application on additional material (DNA isolated from axenic cultures or infected crayfish) revealed that the methods are also useful for characterization of other *A. astaci* genotypes, in which they show marker combinations that differ from those of the reference laboratory strains (Grandjean et al., 2014; Panteleit et al., 2018, 2019; Di Domenico et al., 2021; Mojžišová et al., 2024).

For genotyping purposes, *A. astaci*-positive DNA isolates with agent level A3 (mid-range) or higher were selected. In cases when some genotyping method(s) yielded at least some results but others did not, DNA concentration in the respective isolates was further increased by precipitation with GlycoBlue Coprecipitant (Thermo Fisher Scientific, Waltham, MA, USA), and resuspension of the resulting pellet in a final volume of 20–50 μL.

First, we attempted to amplify fragments of mitochondrial genes for small and large subunits of ribosomal RNA (rnnS and rnnL) according to Makkonen et al. (2018), which were Sanger-sequenced in both directions at the DNA sequencing facility of the Faculty of Science, Charles University, Prague. Obtained sequences were then compared with various *A. astaci* haplotypes documented from the areas invaded by this pathogen, specifically Europe and Japan (Makkonen et al., 2018; Martín-Torrijos et al., 2018) as well as from various parts of North America (Martín-Torrijos et al., 2021b; Martínez-Ríos et al., 2023). The matching haplotypes are indicated by lower-case letters (e.g. ‘a’).

In addition, we applied 5 qPCR assays developed by Di Domenico et al. (2021), which target anonymous genomic regions considered by Minardi et al. (2018) to be diagnostic for *A. astaci* genotype groups. Although more detailed analyses indicated that the identification of *A. astaci* genotypes by these assays is not unambiguous (Di Domenico et al., 2021; Mojžišová et al., 2024), combined information on mitochondrial haplogroup with results of this qPCR genotyping approach substantially increases the resolution compared to using either method alone. The successful amplification of a given assay is indicated by upper-case letters in the results (e.g. ‘B’), the combination of a particular mitochondrial haplotype and qPCR assay for a given sample is separated by slash (e.g. ‘a/C’) throughout the text. The previously studied *A. astaci* genotype groups, originally characterized by random amplified polymorphic DNA (RAPD-PCR; Huang et al., 1994; Diéguez-Uribeondo et al., 1995; Kozubíková et al., 2011a), have the following expected combinations of mtDNA/qPCR genotyping results (Makkonen et al., 2018; Di Domenico et al., 2021): group A: a/A; group B: b/B; group C: a/C; group D: d1/D or d2/D (2 mtDNA haplotypes are known from Europe); group E: e/E.

Finally, for isolates with the highest concentrations of the pathogen DNA, we tried to characterize variation of 9 microsatellite loci developed for *A. astaci* by Grandjean et al. (2014). The original protocol was generally followed, but each locus was amplified and analysed separately, considering that we expected unusual fragment lengths or atypical combinations of alleles. To check for consistency and ensure reproducibility, PCR and fragment analyses were repeated multiple times for any sample and locus that deviated from the others or failed to amplify. In case the results were inconsistent, such loci were conservatively scored as ambiguous and were not considered when interpreting the results. The resulting multilocus genotypes (MLGs) were compared with the reference strains of known *A. astaci* genotype groups (Grandjean et al., 2014, amended in Mojžišová et al., 2020) as well with additional pathogen genotypes previously characterized by these microsatellite markers (Panteleit et al., 2019; Mojžišová et al., 2024).

## Results

## *Distribution and prevalence of* A. astaci

The presence of *A. astaci* was detected at 37 sampling sites (76%; Table 1), in all but 1 crayfish host species (Table 2) and throughout the whole study area (Figures 1 and S1). The exception with no *A. astaci* detection was *Cambarus robustus*, of which only 4 individuals from 3 sites were analysed; in all the other host species studied, the pathogen was detected in the majority of their populations (Table 2, Figure S1). Overall, *A. astaci* was reliably confirmed (with agent level A2 or higher) in 255 out of 533 individuals analysed (48%). When both *A. astaci* detection assays were applied on the same DNA isolates, the detection of the pathogen agreed in all but 1 sample of *F. limosus*, which was very weakly positive with the Vrålstad et al. (2009) assay but not with the Strand et al. (2023) assay; it was conservatively scored as negative, with no influence on population-level results.

The proportion of individuals in which *A. astaci* was detected ranged from 0 to 100% per population, but the confidence intervals of prevalence were wide (Table 1). High *A. astaci* infection rates were found in populations of native hosts (such as the coexisting *C. bartonii* and *F. limosus* in Radley Run) as well as in introduced *F. virilis* or *F. rusticus*. There were a few host populations in which not even traces of *A. astaci* DNA (i.e. agent level A0) were detected; in most of such cases, however, the number of analysed crayfish specimens were too low to conclude that the pathogen was scarce or even absent at those sites (Table 1). The only exception was the *F. virilis* population from Arrowmink Creek in the Delaware basin, from which 30 individuals were analysed without any *A. astaci* detection.

### Pathogen genotyping

At least some *A. astaci* genotyping data were obtained from 24 DNA isolates representing 5 host species (Tables 2 and 3), 2 native ones (*C. bartonii*: 7 individuals, *F. limosus*: 4), 2 introduced ones (*F. rusticus*: 6, *F. virilis*: 6) and 1 host species with uncertain status in the study area (*F. propinquus*: a single individual). For all but one of these samples, one of the 5 qPCR genotyping assays yielded positive results, and for all but 2, partial sequences of at least one of the mitochondrial ribosomal genes were obtained (Tables 1 and 3). The failures to obtain PCR products of the target markers were consistent despite multiple attempts. For 10 isolates, microsatellite genotyping was at least partially successful (consistent scoring of at least 7 out of 9 analysed loci).

**Figure 2.**
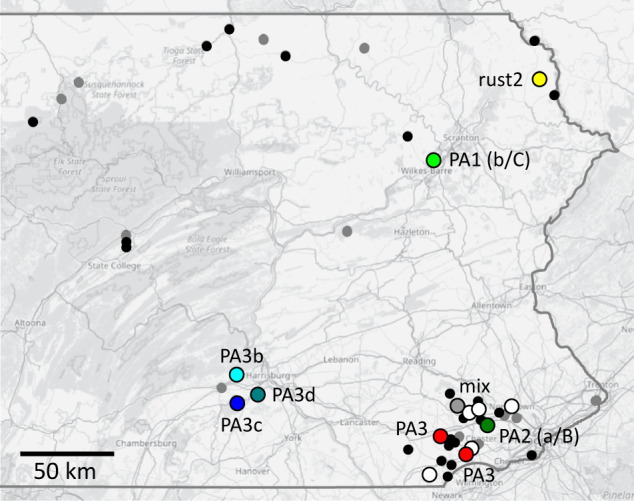
Results of *Aphanomyces astaci* genotyping from host crayfish with a sufficiently high infection load. Small grey dots mark localities with no detection of *A. astaci*, small black dots localities with *A. astaci* presence but no genotyping results. Larger circles indicate sites where genotyping was at least partially successful. White circles mark those where only sequencing of mitochondrial ribosomal marker(s) and/or nuclear qPCR assay were successful. Genotypes with different microsatellite multilocus genotypes (MLGs) are differentiated by colour and MLG code corresponding to Tables 3 and 4. Unless provided otherwise, a combination of mitochondrial haplotype and nuclear qPCR was a/c; the unusual mtDNA/qPCR combinations of MLGs PA1 and PA2 are marked correspondingly.

Most of the genotyped samples (19) yielded a combination of *A. astaci* mitochondrial haplotype a (as revealed by sequences of one of both ribosomal markers) and positive signal of qPCR assay C (a/C hereafter; Table 3; Figure 2). Both DNA isolates for which mtDNA sequencing was unsuccessful were also positive for qPCR assay C. In 1 sample with haplotype a, no qPCR genotyping assay was positive. Two samples, however, stood out due to unusual combinations of mitochondrial haplotype and qPCR genotyping results. A single *A. astaci*-positive specimen of the native crayfish *C. bartonii* from Green Tree Run, Delaware basin, apparently carried an *A. astaci* genotype with mitochondrial haplotype a but positively reacting to qPCR assay B (a/B). Another unusual sample was that from *F. propinquus* collected in Abrahams Creek, Susquehanna basin, which yielded mitochondrial haplotype b (as revealed by both ribosomal markers) but a positive signal from qPCR assay C (b/C).

Results of *Aphanomyces astaci* genotyping in Eastern Pennsylvania

**Table 3.** Detailed results of *A. astaci* genotyping for each analysed crayfish specimen and all 3 complementary methods: sequencing of large and small mitochondrial ribosomal subunits (mtDNA), qPCR-based detection of diagnostic nuclear markers and amplification of 9 microsatellite loci (SSR). Locality numbers correspond to those in Table 1, sample codes match Supplementary Table 1. Allele compositions of microsatellite multilocus genotypes are provided in Table 4

			mtDNA^a^		qPCR^b^		
Locality	Species	Sample	rnnL	rnnS	B	C	SSR MLG
4: West Branch White Clay Creek	*F. limosus*	US-21	a	a	—	39.0	
		US-25	a	a	—	39.1	
		US-27	a	a	—	38.5	
15: North Branch Calkins Creek	*F. rusticus*	USA-36	a	a	—	38.2	rust2
22: Abrahams Creek	*F. propinquus*	USA-86	b	b	—	39.1	PA1
25: trib. to Pickering Creek	*C. bartonii*	USA-112	a	a	—	—	
27: Radley Run	*C. bartonii*	PA-4	N/A	N/A	—	38.9	
28: Bennetts Run	*C. bartonii*	PA-15	a	a	—	35.5	PA3
		PA-16	a	a	—	37.1	PA3
	*F. limosus*	PA-20	a	a	—	38.3	
29: Green Tree Run	*C. bartonii*	PA-24	a	a	36.6	—	PA2
31: Schuylkill River	*F. rusticus*	PA-51	a	a	—	39.5	
34: trib. of West Branch Brandywine Creek	*C. bartonii*	PA-102	a	a	—	36.1	PA3
35: Pine Creek #2	*F. virilis*	PA-117	a	N/A	—	38.5	
		PA-119	a	a	—	37.8	
		PA-122	a	N/A	—	40.0	
42: Birch Run	*C. bartonii*	PA-176	a	a	—	35.9	mix^c^
47: trib. of Conodoguinet Creek	*F. rusticus*	PA-291	a	a	—	38.3	PA3b
		PA-297	a	a	—	39.1	
	*F. virilis*	PA-302	a	a	—	39.1	
		PA-307	a	a	—	38.0	
		PA-315	a	a	—	40.0	
44: Yellow Breeches Creek	*F. rusticus*	PA-216	a	a	—	39.3	PA3c
48: trib. of Yellow Breeches Creek	*F. rusticus*	PA-326	N/A	N/A	—	38.0	PA3d

a N/A indicates consistent failure of amplification of the given marker.

b Numbers indicate cycle threshold for a given qPCR assay (B or C); dashes mark no amplification.

c A consistent amplification of 3 fragments at one of the loci suggests mixed infection (see Table 4).

The unique status of the 2 samples with a/B and b/C marker combinations was also supported by microsatellite genotyping (Tables 3 and 4), which resulted in a combination of allele sizes previously not documented from any other *A. astaci* strains isolated to axenic cultures or genotyped directly from crayfish hosts. Microsatellite MLGs of DNA isolates that yielded haplotype a but qPCR assay C signal (a/C) indicate that these represent more distinct *A. astaci* strains differing at multiple microsatellite loci (Table 4). Three individuals of *C. bartonii* from 2 populations shared the same *A. astaci* MLG (PA3), other MLGs were represented by single DNA isolates. Three of them (PA3b, PA3c, PA3d), from non-native *F. virilis* and *F. rusticus*, shared allele combinations at most loci with PA3 from the native *C. bartonii* but differed in the extent of heterozygosity (Table 4). A MLG labelled ‘rust2’, from *F. rusticus* collected from North Branch Calkins Creek, Delaware basin, was very similar in allele composition to the *A. astaci* ‘rust1-genotype’ characterized from populations of the same species introduced in Wisconsin (Panteleit et al., 2019), differing in allele composition at a single locus Aast9. While a single allele was consistently amplified from the Pennsylvania sample (suggesting a homozygosity at Aast9), the axenic isolates from Wisconsin were heterozygous (Table 4). Lastly, 1 DNA isolate from *C. bartonii* from Birch Run, Delaware basin, consistently yielded 3 peaks at the locus Aast6 (Table 4), likely due to mixed infection by more than 1 *A. astaci* strain (or co-infection with another related oomycete). Interestingly, the allele composition from this sample was otherwise similar to an *A. astaci* MLG recently characterized from established ornamental crayfish in Budapest, Hungary (Mojžišová et al., 2024).

**Table 4.** Characterization of microsatellite multilocus genotypes identified in Pennsylvania, and their comparison with *A. astaci* reference genotypes (based on Grandjean et al., 2014; Panteleit et al., 2019; Mojžišová et al., 2020, 2024). Reference genotype codes refer to culture collections of the Norwegian Veterinary Institute, Oslo (VI) and Finnish Food Authority – Ruokavirasto, Kuopio (Evira)

			SSR locus^a^								
MLG (*host species*)	mtDNA	qPCR	Aast2	Aast4	Aast6	Aast7	Aast9	Aast10	Aast12	Aast13	Aast14
SSR-PA1 (*F. propinquus*)	b	C	162	87	—	215	164/182	132	—	194/202	248
SSR-PA2 (*C. bartonii*)	a	B	?	89	157	207	164/182	132	222	194	—
SSR-PA3 (*C. bartonii*)	a	C	142	87/89	148/154	191	164/182	132/142	222/226	202	248
SSR-PA3b (*F. virilis*)	a	C	142	87	148	191	164/182	132/142	226	202	248
SSR-PA3c (*F. rusticus*)	a	C	142	87/89	148	191	164/182	132/142	226	202	248
SSR-PA3d (*F. rusticus*)	N/A	C	142	87/89	148	191	?	142	226	202	—
SSR-rust2 (*F. rusticus*)	a	C	142	87	148	191	168	132	240	202	248
mix^b^ (*C. bartonii*)	a	C	142/162	87/105	148/154/157^b^	191/207	164/182	132/142	222/226	194/202	248
**Reference genotypes**											
SSR-A1 (Evira6462/06)	a	A	160	103	157	207	180	142	226/240	194	246
SSR-A2 (VI03557)	a	A	160	103	157	207	180	142	—	194	246
SSR-B1 (VI03555)	b	B	142	87	148	215	164/182	132	226/240	202	248
SSR-C (VI03558)	a	C	154	87	148	191	164/168	132	226	202	248
SSR-D1 (VI03556)	d1^c^	D	138	131	148	203	180	142	234	194	250
SSR-E (Evira4805)	e	E	150	87/89	148/157	207	168/182	132/142	240	194/202	248
SSR-rust1	a	C	142	87	148	191	164/168	132	240	202	248
SSR-Up	a	B	142/150	87	148	205/215	164	132/138	226	202	248
SSR-Budapest	a	C	142/162	87/105	148/157	191/207	164/182	132/142	226	194/202	248

a Dashes indicate consistent lack of amplification of the given locus. Question marks (for MLGs PA2 and PA3d) indicate loci that could not be unambiguously scored.

b A consistent amplification of 3 fragments at the locus Aast6 suggests the presence of more than 1 *A. astaci* strain or more than 1 oomycete species.

c Mitochondrial haplotype has not been determined for this particular reference strain but this haplotype is most likely, considering its distribution across Spain (Martín-Torrijos et al., 2019).

In 2 cases, *A. astaci* genotyping from different host species coexisting in the same streams was possible: from native *C. bartonii* and *F. limosus* in Bennets Run, Delaware basin, and from introduced *F. rusticus* and *F. virilis* in an unnamed tributary of Conodoguinet Creek, Susquehanna basin. All those crayfish yielded the same a/C combination of mtDNA/qPCR markers, but microsatellite analysis was only successful for one of the 2 syntopic hosts (Table 3), so it was not possible to assess whether the 2 coexisting species shared the same *A. astaci* genotype.

## Discussion

Our study, based on comparable methods to those repeatedly used to screen for *A. astaci* in the regions invaded by this pathogen, confirmed that both population-level prevalences and individual pathogen loads in cambarid crayfish in the USA are similar to those in Europe (see references below) and Japan (Mrugała et al., 2017). Crayfish native as well as introduced to Pennsylvania carry *A. astaci* frequently; the pathogen was widespread in the study region, and except of 1 site (30 negative-testing non-native *F. virilis* from Arrowmink Creek), we cannot claim with confidence that *A. astaci* prevalence in any population was low. The pattern that populations from nearby water bodies show contrasting infection rates is common (e.g. Laffitte et al., 2024), and cambarid crayfish also tend to have relatively low infection loads (usually not exceeding agent level A4, i.e. moderate levels of *A. astaci* DNA) in their invaded ranges outside of North America (Tilmans et al., 2014; Mrugała et al., 2017; Mojžišová et al., 2022, 2024; Laffitte et al., 2024).

Strongly infected North American hosts with agent levels A5 and higher have only occasionally been reported in studies focusing on *A. astaci* screening. Such high infection loads, observed for example in *P. clarkii* in Brazil (Peiró et al., 2016), *F. rusticus* in Wisconsin (Panteleit et al., 2019), *P. clarkii* in France (Laffitte et al., 2024) and *P. virginalis* in Hungary (Mojžišová et al., 2024), are comparable to samples from susceptible crayfish hosts affected by acute crayfish plague (e.g. Vrålstad et al., 2014; Caprioli et al., 2018) and indicate extensive growth of the pathogen in host tissues. Such DNA isolates are particularly suitable for *A. astaci* genotyping. In samples from Pennsylvania, we did not encounter any specimen with high agent levels. However, genotyping *A. astaci* from DNA isolates with agent level A4 (moderate; exceeding 1000 PFU in qPCR assay targeting the ITS marker) by the methods used in our study is usually successful (Grandjean et al., 2014; Makkonen et al., 2018; Di Domenico et al., 2021). Even some samples with agent level A3 (below 1000 PFU) tend to yield positive results (especially when additional steps to increase DNA concentration in the isolates are applied; Mojžišová et al., 2022), although microsatellite genotyping (Grandjean et al., 2014) clearly requires higher concentration of pathogen DNA than either mitochondrial haplotyping (Makkonen et al., 2018) or the qPCR genotyping assays (Di Domenico et al., 2021).

Patterns of microsatellite variation from mixed genomic samples must be considered with caution, as not all loci characterized by Grandjean et al. (2014) are specific for *A. astaci* only, and coinfections by different strains of the pathogen (Mojžišová et al., 2024) or by *A. astaci* and another oomycete may lead to unusual combination of detected allele sizes (as was also the case in one of our samples; Table 3). Furthermore, amplification of microsatellite loci from DNA isolates with low concentration of pathogen DNA may be inconsistent or fail entirely. However, when distinct microsatellite MLGs match other genotyping assays (as was the case in our MLGs PA1 and PA2 with b/C and a/B marker combination), or when corresponding alleles are observed in multiple host individuals (such as our MLG PA3), the results may be considered conclusive, and the use of these markers provides important additional insight into the pathogen variation.

Small differences characterized only by differing levels of heterozygosity, as observed between MLGs PA3 to PA3d, or between Pennsylvanian rust2 and reference genotype rust1 from *F. rusticus*, might possibly result from inconsistent amplification of low-concentration templates, so we cannot entirely rule out that these in fact represent the same *A. astaci* genotypes. However, it should be noted that the variation of the respective loci was consistent across multiple polymerase chain reactions and fragment analyses, and such minor differences are comparable with already documented microsatellite MLG variation within *A. astaci* genotype groups (Grandjean et al., 2014; Maguire et al., 2016; James et al., 2017; Mrugała et al., 2017). Interestingly, Butler et al. (2020) also reported minor variation (in homo- vs heterozygosity) in sequences of a gene for chitinase among 4 *A. astaci* strains isolated from different *F. obscurus* host specimens originating from the same population.

Our results confirm that studies focusing on diversity of *A. astaci* should combine, whenever possible, different available genotyping methods to provide more detailed insight into the patterns of variation of this pathogen. This is well illustrated when contrasting the results of mtDNA sequencing and qPCR genotyping based on nuclear markers. Each of these methods independently indicated the presence of at least 2 different *A. astaci* genotypes in our samples, and each of them pointed to the potential presence of *A. astaci* genotype group B, documented so far only from *P. leniusculus* in the western USA (Makkonen et al., 2019) and from regions where this host species was introduced (Martín-Torrijos et al., 2018; Ungureanu et al., 2020). However, when the markers were evaluated together, we observed 3 distinct mtDNA/qPCR combinations (frequent a/C, and rare a/B and b/C), none of which matched *A. astaci* group B (with expected b/B pattern: Makkonen et al., 2018; Di Domenico et al., 2021). The distinctness of the 2 unusual *A. astaci* genotypes was also supported by microsatellite markers, which additionally indicated that the genotyped samples from Pennsylvania pooled under a/C comprised more strains.

Neither the 2 unique isolates with a/B and b/C genotyping combinations, nor the other samples characterized by microsatellite markers, corresponded to any *A. astaci* genotype known so far. The a/B marker combination has been documented from Europe, from the genotype ‘Up’ causing mass mortalities of native crayfish in Czechia (Kozubíková-Balcarová et al., 2014; Mojžišová et al., 2020) but also from a chronic infection of a narrow-clawed crayfish *Pontastacus leptodactylus* in the Danube (Panteleit et al., 2018). Grandjean et al. (2014) speculated, based on allele composition, that this genotype may have been introduced to Europe with *P. leniusculus* (i.e. a host originating from the Pacific drainages of North America). The a/B sample from a cambarid host from Pennsylvania, *C. bartonii*, yielded distinctly different microsatellite MLG (Table 4), so it does not seem closely related to the ‘Up’ genotype.

The b/C marker combination is new for *A. astaci*. Our genotyped sample from *F. propinquus* represents the first documented presence of *A. astaci* mitochondrial haplotype b in eastern North America (see Martín-Torrijos et al., 2021b). Both qPCR genotyping and microsatellite MLG indicate that this is not any genotype known from *Pacifastacus*. Horizontal transmission of *A. astaci* genotype group B from this host to cambarids has been documented from the contact of invasive crayfish in Europe, either from signal crayfish (James et al., 2017; Mojžišová et al., 2022) or between various cambarids, some of which originated from ornamental aquaria (Mojžišová et al., 2024). In none of those cases, however, the marker combination was close to that from Pennsylvanian *F. propinquus*. We assume that this *A. astaci* genotype may be natively occurring in cambarids. The unusual combination of mitochondrial haplotypes and nuclear markers raises a question about past evolution and dispersal of *A. astaci* strains, and potential gene flow between them, considering that sexual reproduction has not been documented from this pathogen (Diéguez-Uribeondo et al., 2009; Rezinciuc et al., 2015; Martínez-Ríos et al., 2023). As Rezinciuc et al. (2015) speculated, it is possible that strains studied so far, in most cases isolated from *A. astaci*’s invaded range, represented the same mating type. It might be thus worth exploring whether the reproductive biology of this pathogen in its native range differs from that in Europe. It is noteworthy, however, that sexual structures are not known for most *Aphanomyces* species parasitizing animals (Diéguez-Uribeondo et al., 2009).

The combination of mitochondrial and nuclear markers a/C, documented by us from multiple species, both native (*C. bartonii, F. limosus*) and invasive (*F. rusticus, F. virilis*) corresponds to that reported from *A. astaci* strains isolated from the locally non-native *F. obscurus* collected from one site in Pennsylvania by Butler et al. (2020). However, the same marker combination was also observed in *A. astaci* genotype group C (a strain isolated from *P. leniusculus* originating from British Columbia; Huang et al., 1994), in another strain ‘rust1’ isolated from invasive populations of *F. rusticus* from Wisconsin (Panteleit et al., 2019), and in an *A. astaci* genotype introduced, apparently with ornamental cambarid crayfish, to Budapest, Hungary (Mojžišová et al., 2024). None of the Pennsylvania samples genotyped by microsatellites corresponded to genotype group C, as originally defined by Huang et al. (1994) by RAPD-PCR. One MLG characterized by us from *F. rusticus*, however, had an allele composition very similar, although not identical, to a genotype isolated from the same host elsewhere (Panteleit et al., 2019), which may indicate that this invasive crayfish contributes to dispersal for several related *A. astaci* genotypes. However, our study indicates that even at a limited spatial scale, the strain variation of *A. astaci* within both native (*C. bartonii*) and invasive (*F. rusticus*) cambarid crayfish may be substantial.

We failed to amplify some of the diagnostic markers (mitochondrial ribosomal subunits or any of the nuclear markers targeted by qPCR assays) from several samples. Specifically, no qPCR assay was positive for 1 sample from infected *C. bartonii*. We do not know whether this represents an infection by a distinct *A. astaci* genotype that lacks all of the target regions in its genome or simply an amplification failure. Considering a relatively low amount of pathogen DNA in that sample, the latter explanation is possible although its mtDNA sequencing was successful. However, the existence of ‘non-amplifying’ *A. astaci* strains is also very likely. In fact, we found it surprising that the assays based on anonymous nuclear markers, primarily developed for fast screening for the common *A. astaci* genotypes causing mass mortalities in Europe (Di Domenico et al., 2021), turned out to be usable in the native range of this pathogen, where substantially higher intraspecific variation even at a small regional scale would be expected (Martín-Torrijos et al., 2021b).

An occasional repeated failure to amplify one of the mtDNA markers (attempted multiple times) could have been caused by previously undetected intraspecific variation in the primer region that was designed to avoid, as much as possible, amplification of other oomycetes (Makkonen et al., 2018). Indeed, consistent failure to amplify the large mitochondrial ribosomal subunit was reported from *A. astaci* infecting *P. clarkii* hosts in Costa Rica (Martín-Torrijos et al., 2021a). In 2 cases, when none of the mitochondrial markers amplified, and thus no sequence-based confirmation of the species identity was available, the observed patterns might also be explained by an undetected presence of another, closely related *Aphanomyces* species that cross-reacts with the ITS-based qPCR assay of Strand et al. (2023). This is not impossible, considering the limited knowledge of *Aphanomyces* spp. that occur on American crayfish (but see Kozubíková-Balcarová et al., 2013; Makkonen et al., 2019; Butler et al., 2020) or in their environments, and also in the light of recent discovery of *A. fennicus*, a species closely related to *A. astaci* but apparently avirulent to crayfish (Viljamaa-Dirks and Heinikainen, 2019). *Aphanomyces fennicus* cross-reacted with a previously used detection assay of Vrålstad et al. (2009) but also with one of the nuclear qPCR genotyping assays of Di Domenico et al. (2021). A scenario of other cross-reacting species presence seems feasible in the case of *C. bartonii* from Bradly Run (sample PA-4, Table 3), less so for *F. rusticus* from the tributary of Yellow Breeches Creek (sample PA-326), for which 7 microsatellite markers were scored, with allele sizes overlapping with the MLG PA3 repeatedly scored from *C. bartonii* (Table 4).

As pointed out above, genotyping *A. astaci* from mixed-genome samples, isolated directly from chronically infected hosts, is challenging due to low concentration of target DNA as well as potential cross-amplification of non-target taxa. To avoid methodological biases and to better characterize *A. astaci* diversity from its native range, DNA isolates from axenic cultures obtained from infected hosts (such as those from Panteleit et al., 2019; Butler et al., 2020; Martín-Torrijos et al., 2021a, 2021b) should be studied in more detail, and by multiple genotyping methods. Furthermore, alternative variable markers to microsatellites (e.g. single nucleotide polymorphism arrays), optimized for genotyping from mixed genome samples with low *A. astaci* agent levels, could be developed. While the presently available microsatellite markers are convenient when genotyping the pathogen in laboratory cultures or in crayfish plague outbreaks of susceptible host species (e.g. Grandjean et al., 2014; Vrålstad et al., 2014; Kaldre et al., 2017), the success is substantially lower for hosts that act as *A. astaci* asymptomatic carriers and tend to have much lower infection loads.

To conclude, our study confirmed that *A. astaci* infections are common across the study area and in most studied host crayfish species, regardless of their native or invasive status in Pennsylvania. We revealed several yet unknown *A. astaci* genotypes (characterized by multiple marker combinations). Some of the crayfish taxa apparently host more than 1 *A. astaci* genotype in the study region. Considering the number of host species and populations screened, however, the observed *A. astaci* variation, especially at the level of mitochondrial haplotypes, was lower than we expected. Specifically, we failed to find genotype group E introduced with *F. limosus* to Europe; all 4 infected specimens of this host species, from which *A. astaci* could be genotyped, carried another strain. Apparently, *A. astaci* group E, if present in Pennsylvania, is not particularly common in the region studied by us. It may have declined with *F. limosus*, a species that is a widespread invader in Europe (Kouba et al., 2014) but has been replaced at many localities in Pennsylvania by introduced crayfish species (Lieb et al., 2011b). *A. astaci* group E could have also been locally displaced by some other, more successful strain (as recorded in coexisting non-native cambarid crayfish in Budapest; Mojžišová et al., 2024), possibly even one introduced by some of the invasive crayfish taxa in the region. Most likely, however, this particular *A. astaci* genotype has a spatially restricted distribution and might eventually be found in other parts of *F. limosus* range. If distributed over a limited area only, the presence of this genotype may even indicate the potential source region for its host’s introduction to Europe.

## Supporting information

Petrusek et al. supplementary material 1 Petrusek et al. supplementary material

Petrusek et al. supplementary material 2 Petrusek et al. supplementary material

## Supplementary material

The supplementary material for this article can be found at https://doi.org/10.1017/S0031182025000022.
