# Supplementary material for: Prevalence and diversity of Aphanomyces astaci in cambarid crayfish of Pennsylvania: where native and introduced hosts meet
Source: Parasitology. 2025 Jan 23;152(1):92–105. doi: 10.1017/S0031182025000022 (PMC12088921; doi:10.1017/S0031182025000022)

Supplementary Fig. S1. Distribution of sampled crayfish populations and their *Aphanomyces astaci* infection status in Pennsylvania. The size of the symbols is proportional to the number of crayfish analysed, the fill colour indicates the prevalence of *A. astaci*. Grey dots are localities from which no specimen of the given species was analysed.

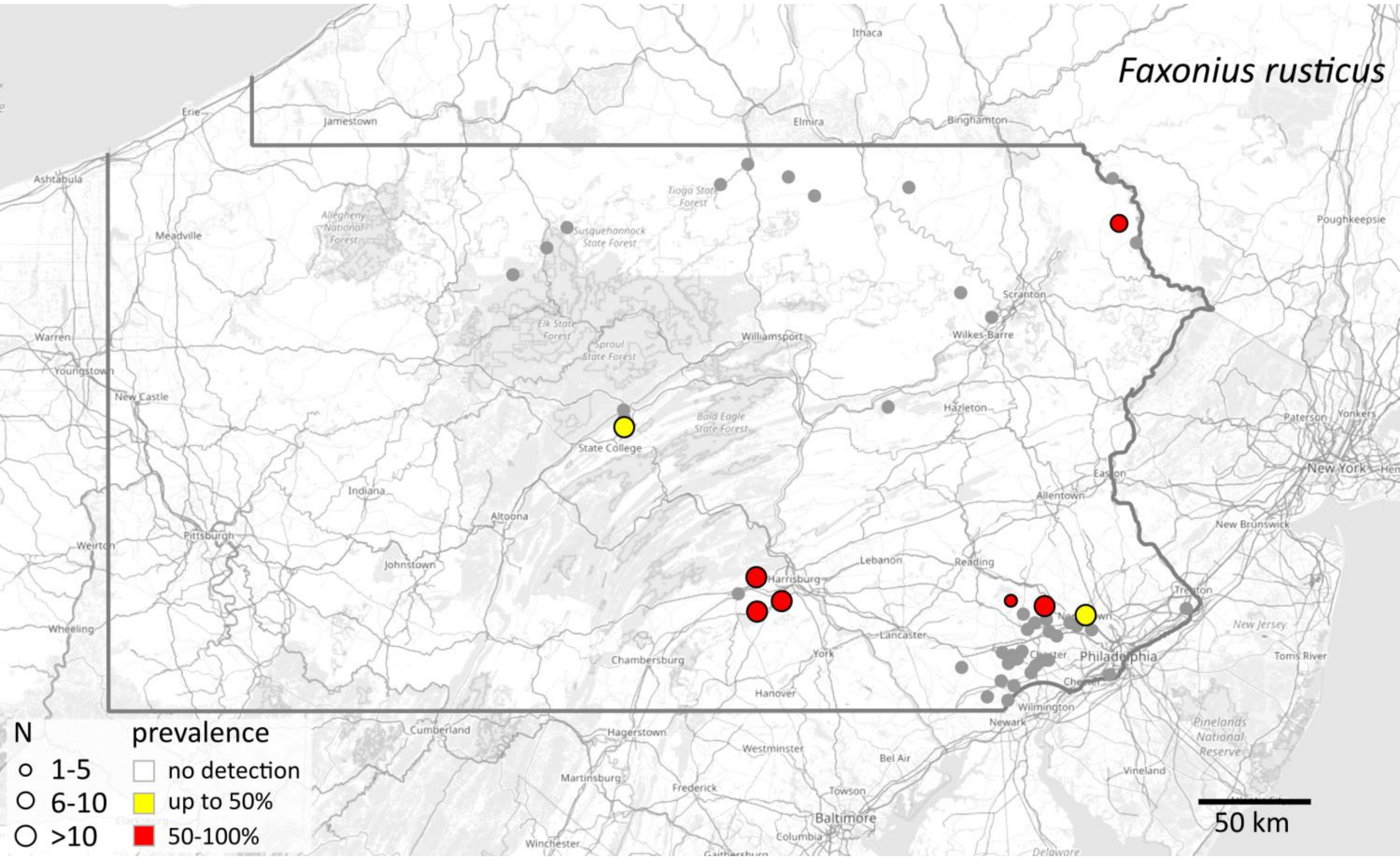

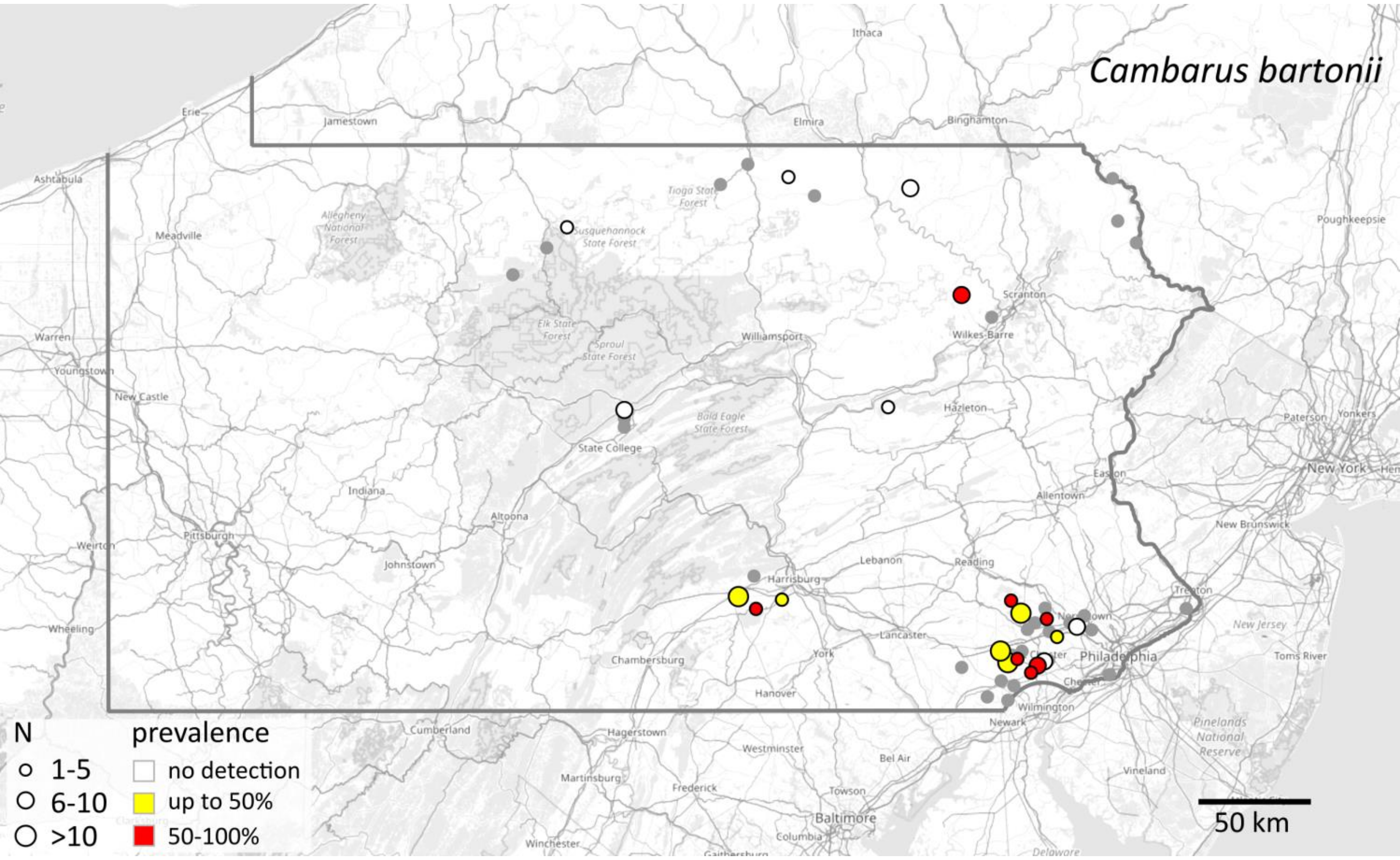

Supplementary Fig. S1 (continued).

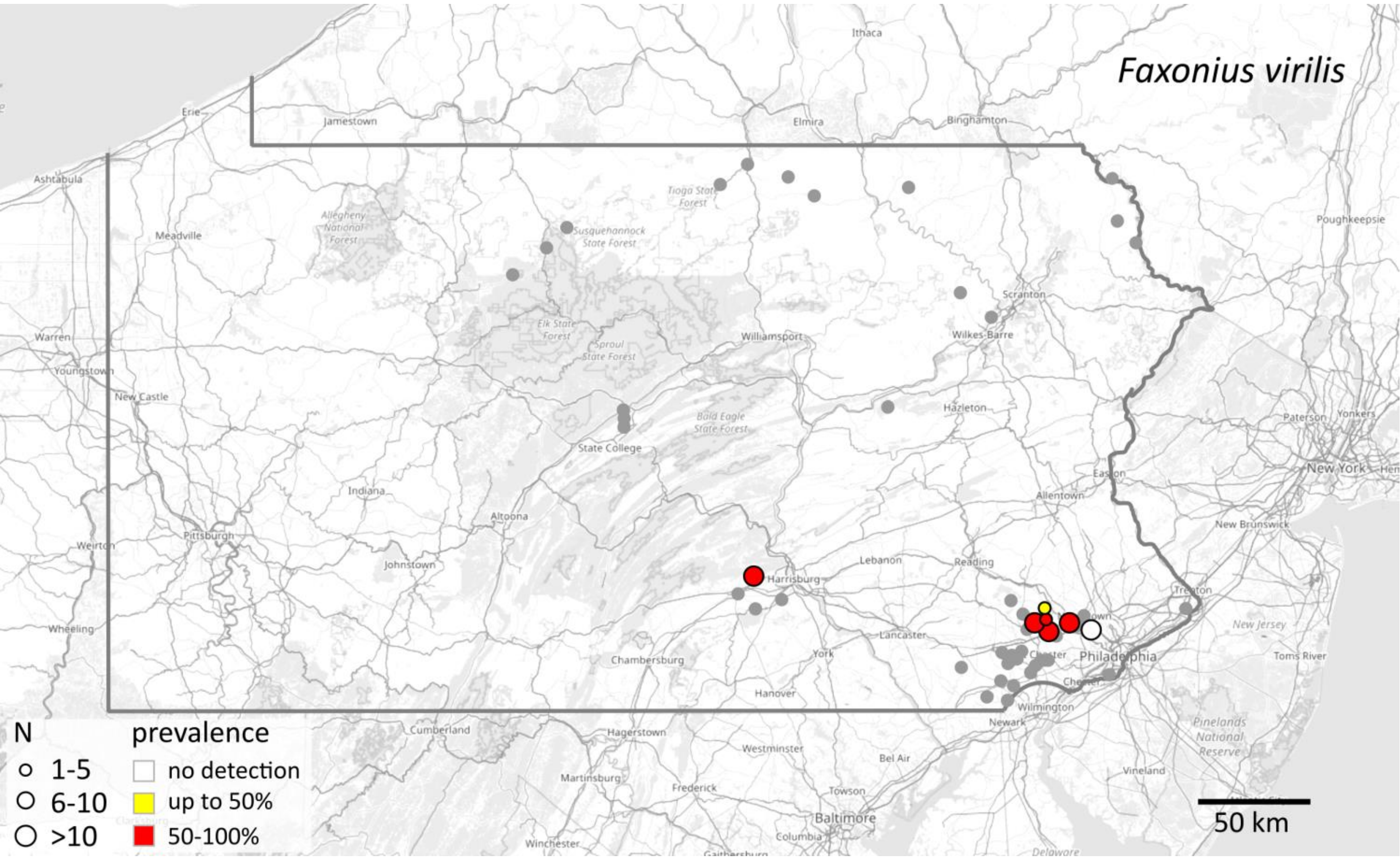

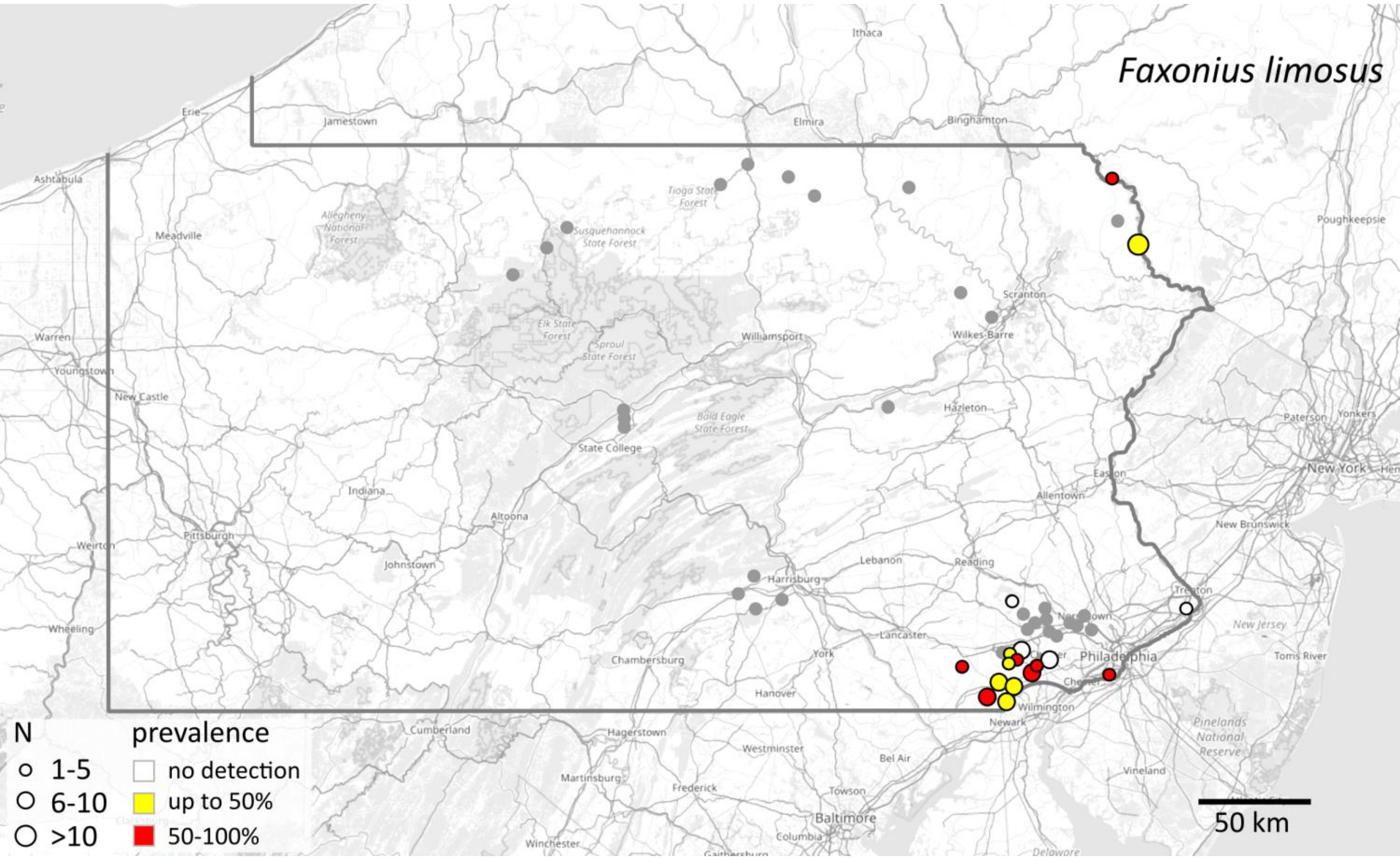

Supplementary Fig. S1 (continued).

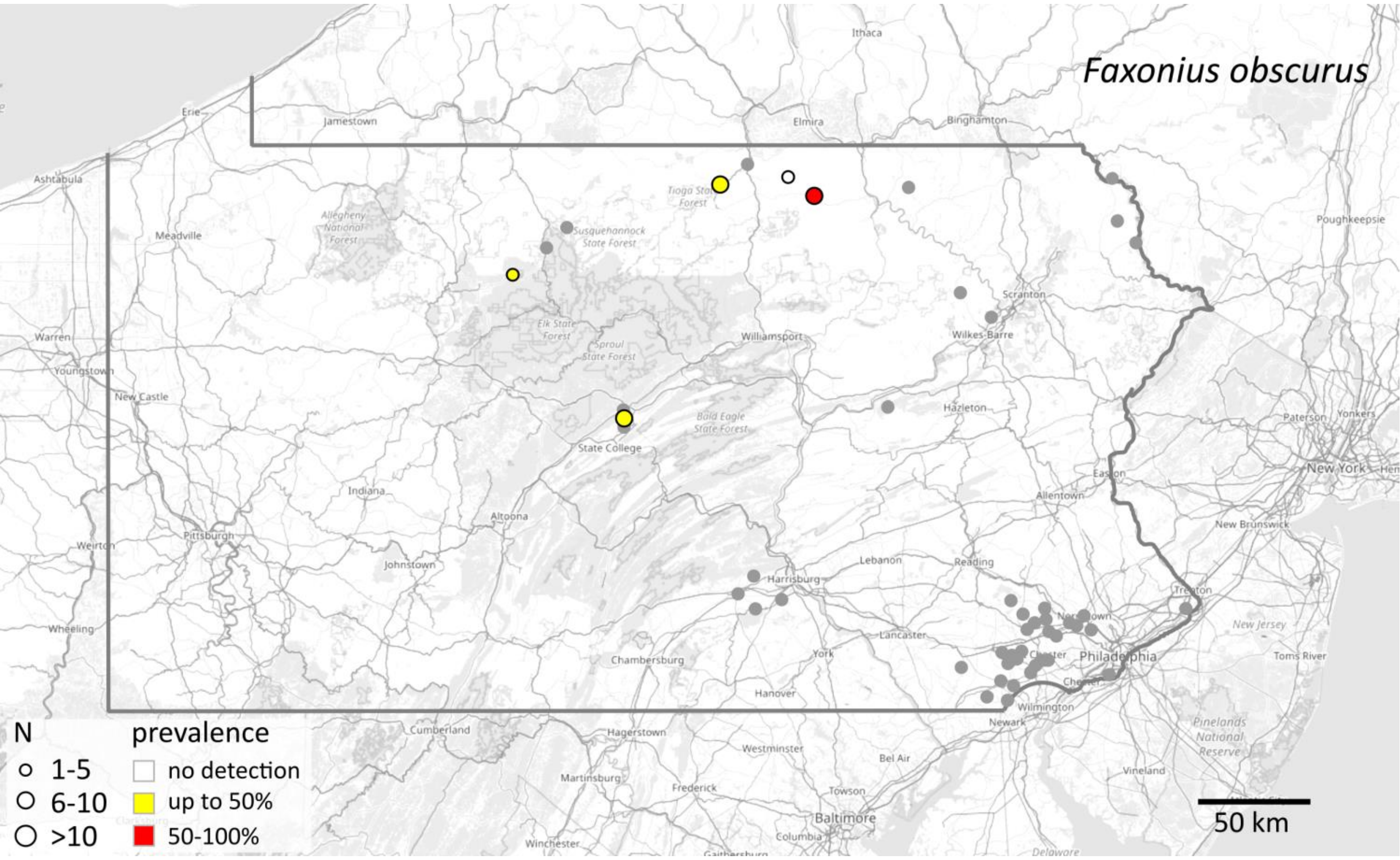

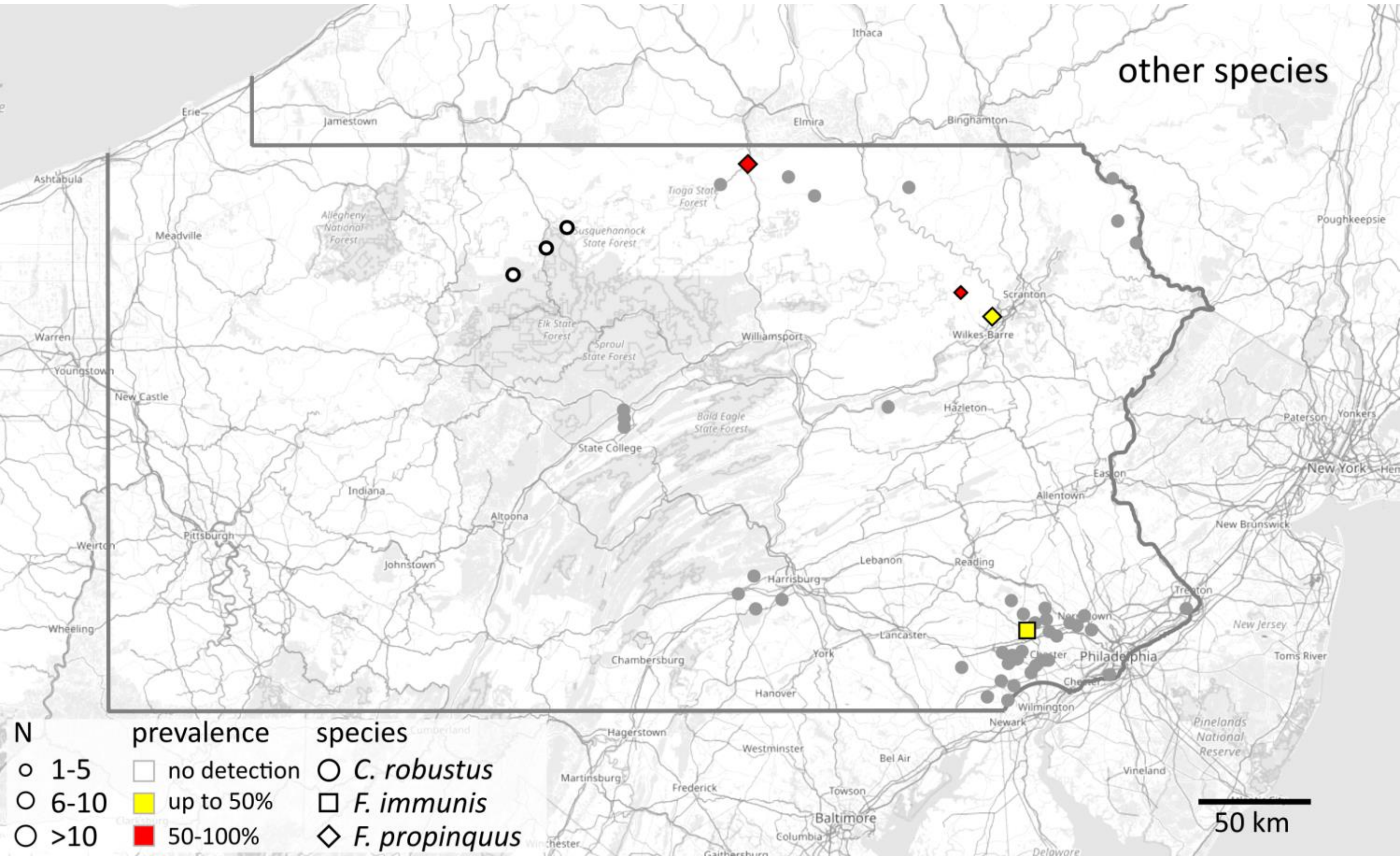

Supplement: Petrusek et al. supplementary material 1 — Petrusek et al. supplementary material [file S0031182025000022sup001.pdf]
